# In vitro characterization and inhibition of the CXCR4/CXCL12 chemokine axis in human uveal melanoma cell lines

**DOI:** 10.1186/1475-2867-7-17

**Published:** 2007-11-14

**Authors:** Sebastian Di Cesare, Jean-Claude Marshall, Bruno F Fernandes, Patrick Logan, Emilia Antecka, Vasco Bravo Filho, Miguel N Burnier

**Affiliations:** 1 The Henry C. Witelson Ophthalmic Pathology Laboratory and Registry, McGill University Health Center, Montreal, PQ, Canada; 2 Department of Ophthalmology, Federal University of São Paulo – UNIFESP/EPM, São Paulo, Brazil

## Abstract

**Purpose:**

The CXCR4/CXCL12 chemokine axis may play a critical role in guiding CXCR4+ circulating malignant cells to organ specific locations that actively secrete its ligand CXCL12 (SDF-1) such as bone, brain, liver, and lungs. We sought to characterize the presence of the CXCR4/CXCL12 axis in five uveal melanoma (UM) cell lines in vitro. The ability of TN14003, a synthetic peptide inhibitor that targets the CXCR4 receptor complex, to inhibit this axis was also assessed.

**Methods:**

Immunocytochemistry was performed against CXCR4 to confirm expression of this chemokine receptor in all five UM cell lines. Flow cytometry was preformed to evaluate CXCR4 cell surface expression on all five UM cell lines. A proliferation assay was also used to test effects TN14003 would have on cellular proliferation. Inhibition of cellular migration by specifically inhibiting the CXCR4/CXCL12 axis with TN14003 was also investigated. The binding efficacy of TN14003 to the CXCR4 receptor was assessed through flow cytometric methods.

**Results:**

The CXCR4 receptor was present on all five UM cell lines. All five cell lines expressed different relative levels of surface CXCR4. TN14003 did not affect the proliferation of the five cell lines (p > 0.05). All cell lines migrated towards the chemokine CXCL12 at a level greater than the negative control (p < 0.05). All 5 cell lines pre-incubated with TN14003 prevented cellular migration towards chemokine CXCL12 (p < 0.01). TN14003 preferentially binds CXCR4 to native ligand CXCL12.

**Conclusion:**

Interfering with the CXCR4/CXCL12 axis, using TN14003 was shown to effectively down regulate UM cell migration in vitro. Knowing that UM expresses the CXCR4 receptor, these CXCR4+ cells may be less likely to colonize distant organs that secrete the CXCL12 ligand, if treated with an inhibitor that binds CXCR4. Further studies should be pursued in order to test TN14003 efficacy in vivo.

## Introduction

Uveal melanoma is the most common primary intra-ocular malignant tumor in adults. Despite advances in diagnosis and local treatment of patients over the last 30 years, the mortality rate has remained constant, with 30 to 50% of patients developing metastatic disease [1]. Due to the lack of lymphatics in the eye, metastasis of uveal melanoma occurs via hematogenous dissemination, with most metastases developing in the liver [2].

Metastatic disease is typically characterized by initial cell transformation within the primary tumor site followed by angiogenesis, facilitating nutrient delivery to the newly transformed cells. Later events include cellular proliferation and detachment from the local extracellular matrix, followed by cell migration and intravasation into the newly formed vessels [3]. Interactions between tumor cells and the local environment containing secreted chemokines are thought to be important in a number of tumorigenic processes including the initial cell migration and intravasation. Therefore, in vitro studies investigating these interactions are important to further our understanding of the steps involved in the metastatic cascade.

It is now well described and understood that chemo-attractive cytokines referred to as chemokines play a number of functional roles in tumor biology [4-8]. Chemokines are known to mediate leukocyte trafficking, haematopoiesis, inflammation, angiogenesis, and tumorigenesis [5]. Recent evidence has demonstrated that chemokines play a critical role in cellular transformation, tumor growth, homing, and metastasis [9].

These low molecular weight signaling molecules regulate and direct circulating immune system cells to specific locations in the body. It has also recently been discovered that cancer cells, themselves exploit and "hijack" the chemokine system in order to facilitate their movement, and extravasate out of the primary site into systemic circulation [6].

It is widely believed that the CXCR4/CXCL12 axis may play a critical role in guiding circulating malignant cells (CXCR4+ cells) to organ specific locations that actively secrete CXCL12 (SDF-1) such as bone, brain, liver, and lungs [10]. Many animal models have also shown very impressive results, while inhibiting the CXCR4/CXCL12 axis, significantly inhibiting and delaying the metastatic process [11,12]. It has also been recently established that high CXCR4 positivity in primary human uveal melanoma specimens correlates with cell type, which is a well established prognostic indicatior for the development of metastatic uveal melanoma [13]. Our laboratory has previously shown that uveal melanoma cellular migration can be induced in vitro when exposing uveal melanoma cell lines to exogenous sources of CXCL12 [14]. We have also shown that uveal melanoma cell lines (92.1, SP6.5, MKT-BR, OCM-1, UW-1) do not express any detectable levels of CXCL12 when analyzed with quantitative real-time PCR [14]. This is important because epigenetic silencing of CXCL12 in human colorectal carcinoma cell lines has shown to promote and even enhance tumor metastasis in vitro and in vivo [15]. These results suggest that when autocrine signaling in the CXCR4/CXCL12 axis is disrupted, tumor cells become more "malignant" due to the fact that they can more readily sense CXCL12 secreted from distant sites [15].

We have shown that interfering with the CXCR4/CXCL12 axis using TN14003 in vitro will effectively inhibit tumor cell migration. Further in vivo studies are warranted to test the efficacy of TN14003 to inhibit UM metastasis.

## Materials and methods

### Cell culture

Four human UM cell lines (92.1, SP6.5, MKT-BR, OCM-1) and one transformed melanocyte cell line (UW-1) were incubated at 37°C in a humidified 5% CO_2 _enriched atmosphere. These cells were cultured with RPMI-1640 medium (Invitrogen), supplemented with 5% heat inactivated fetal bovine serum (FBS; Invitrogen), 1% fungizone (Invitrogen), and 1% penicillin twice weekly, at every media change, for normal growth by phase contrast microscopy. The cultures were grown to confluence and passaged by treatment with 0.05% trypsin in EDTA (Corning) at 37°C and washed in 7 ml RPMI-1640 media before being centrifuged at 120 g for 10 minutes to form a pellet.

The UM cell lines 92.1, SP6.5, MKT-BR, OCM-1, and UW-1 had been established by Dr. Jager (University Hospital Leiden, The Netherlands), Dr. Pelletier (Laval University, Quebec, Canada), Dr. Belkhou (CJF INSERM, France) and Dr. Albert (University of Wisconsin-Madision, USA), respectively [16,17].

### Synthetic peptide inhibitor

The CXCR4 synthetic peptide inhibitor was synthesized and purchased from the Emory University Microchemical Facility, Winship Cancer Center. The peptide itself is approximately 2.03 kDa in size and its linear amino acid sequence is as follows: NH2-RR-Nal-CY-Cit-K-dLys-PYR-Cit-CR-amide.

### Immunocytochemistry

Cytopsins of the 5 UM cell lines were made using a Cytospin3 machine (Shandon). Cells from culture were diluted to a concentration of 250,000 cells/ml, and a 300 μL solution at that concentration was placed in each spin to be evenly plated on each slide. All slides were then immunostained with primary anti-human monoclonal antibody against CXCR4 (R&D Systems, clone #: 12G5) using the Ventana™ automated immunostaining machine programmed to use a standard Avidin-Biotin Complex method.

### Proliferation assay

The Sulforhodamine-B based assay kit (TOX-6, Sigma-Aldrich, St. Louis, Missouri, USA) was performed according to the National Cancer Institute protocol [18]. Each of the 5 UM cell lines (92.1, SP6.5, MKT-BR, OCM-1, UW-1) was seeded into wells at a concentration of 5.0 × 10^3 ^cells per well, with twelve wells per cell line. A row of twelve wells containing only RPMI-1640 medium was used as a control. Cells were allowed to adhere overnight. The CXCR4 peptide inhibitor TN14003 was added the previous day to six of the twelve wells per cell line at a concentration of 4 ng/ml [12]. Cells were then allowed to incubate for 48 hours following the addition of TN14003. Following this 48 hour period, cells were fixed to the bottom of the wells using a solution of 50% Trichloroacetic acid (TCA) for 1 hour at 4°C. Plates were then rinsed with distilled water, to remove the TCA and excess media, then the plates were air dried. The Sulforhodamine-B dye solution was then added to each well and allowed to stain for 25 minutes. The Sulforhodamine-B solution was subsequently removed by washing with a 1% acetic acid solution and once more allowed to air dry. The dye that had become incorporated into the fixed cells at the bottom of the wells was solubilized in a 10 mM solution of Tris. The absorbance of the solute was measured using a microplate reader at a wavelength of 565 nm.

### Cell migration assay

The migratory and inhibitory properties of 5 UM cell lines (92.1, MKT-BR, OCM-1, SP6.5, and UW-1) for the CXCR4/CXCL12 chemokine axis were investigated using a QCM™ 24-Well Colorimetric Cell Migration Assay (Chemicon). This assay is based on the Boyden chamber system that contains smaller well inserts. These well inserts have a PET membrane made of a polycarbonate material perforated with 8 μm pores. 5.0 × 10^5 ^cells were then placed in the upper chamber and re-suspended in RPMI serum-free media. The lower chamber contained CXCL12 (SDF-1α) also re-suspended in serum free media at a pre-determined optimum concentration range 200 ng/ml [12]. Test cells were pre-incubated with TN14003 a 2.04 kDa CXCR4 peptide inhibitor at a pre-determined concentration range of 4 ng/ml [12], before being placed in the upper chamber of the migration assay. Appropriate positive and negative controls (10% FBS [+], Serum Free Media [-]) were also used. These plates were incubated for 24 h at 37°C in a 5% CO_2 _supplemented atmosphere. Following incubation, cells that did not migrate were removed from the top of the insert with sterile cotton swabs. Cells that migrated were then stained, eluted from the bottom of the wells, and the optical density of the stain released from the well was read at an O.D of 565 nm.

### Flow cytometry

The CXCR4 cell surface expression was measured and quantified using a Flurokine: Cytokine Flow Cytometry Kit for human SDF-1α Biotin Conjugate (R&D Systems). Cells from culture were scrapped from the bottom of each flask in order to avoid cell surface receptor damage due to trypsinization of adherent cells. Once scrapped, cells were washed twice in 10 mM HBSS (invitrogen), centrifuged at 500 × g for 5 minutes to avoid any cross reactivity with endogenous cytokine secretions. Preceding the washes, cells were counted and seeded in 1.5 ml tubes at a final concentration of 4 × 10^6 ^cells/ml. 10 μL of the biotinylated cytokine reagent was added to each vial containing the cell lines (92.1, SP6.5, MKT-BR, OCM-1, UW-1), 10 μL of biotinylated negative control reagent was added to separate vials containing each of the 5 cells lines as an appropriate negative control for this experiment. All vials were incubated for 60 minutes at 5°C as per protocol instructions. Then 10 μL of the avidin-FITC reagent was added to each tube and were incubated for 30 min. at 5°C in the dark as per protocol instructions. The vials containing the cell reaction mixture were then washed twice with 2 mL of RDF1 buffer provided with the kit in order to remove unreacted avidin-fluorescein. The cells were then finally resuspended in 200 μL of RDF1 buffer for flow cytometric analysis.

### Statistical analysis

Results from the migration assay for each cell line (cells incubated with TN14003) were directly compared to a matched group of cells incubated solely with CXCL12 using a Student's t-test. A significant result was considered when a p-value < 0.05 was obtained. Results from the proliferation assay for each cell line (cells incubated with TN14003) were directly compared to a matched group of cells that did not contain any inhibitor using a Student's t-test. A significant result was considered when a p-value < 0.05 was obtained.

## Results

### Immunocytochemistry

**Figure 1:**
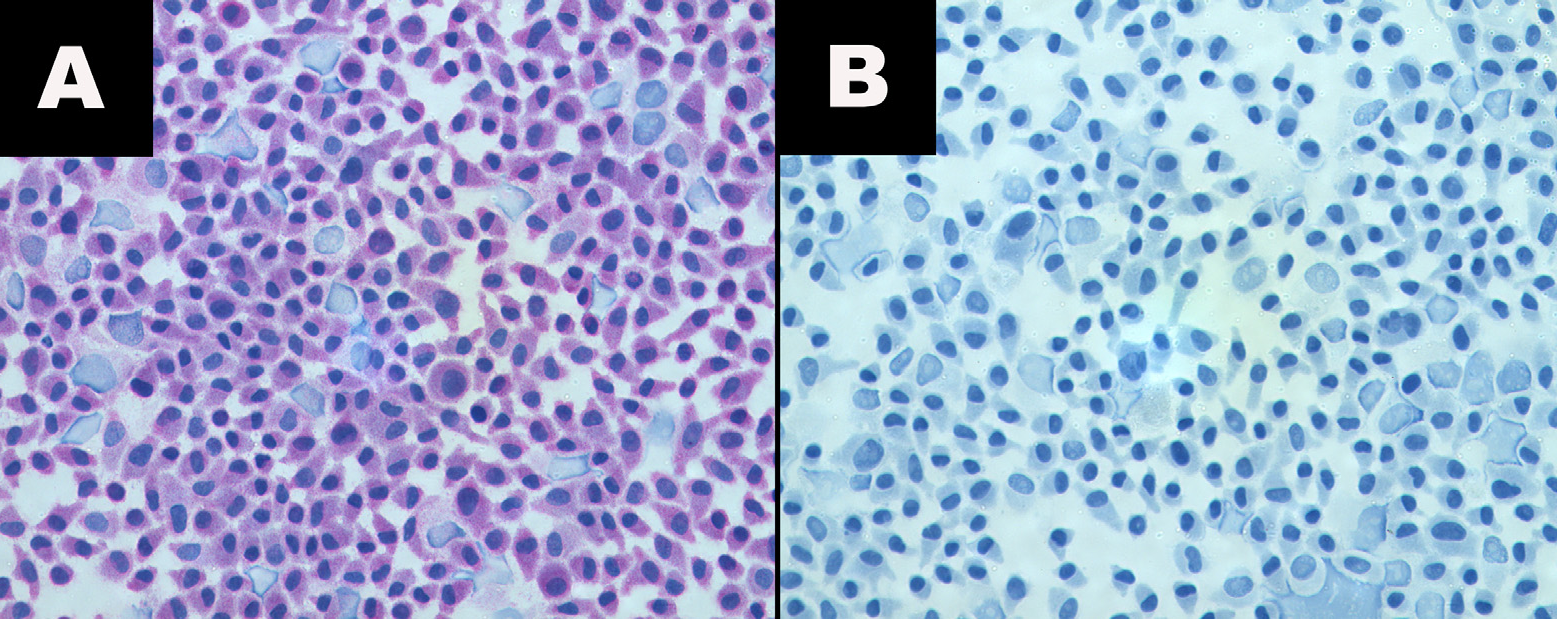
**Immunocytochemistry for CXCR4**. a) Photomicrograph of the MKT-BR cell line (cytospin) stained for CXCR4 (400X) displaying mainly cytoplasmic expression of the receptor. Mild nuclear staining is also evident in this image. b) Photomicrograph of the negative control for the MKT-BR cell line (400X).

All 5 UM cell line cytospins (92.1, SP6.5, MKT-BR, OCM-1, UW-1) stained positive for the CXCR4 receptor. All the slides displayed cytoplasmic staining of the receptor in the cell lines when viewed under a light microscope at a magnification of 400x (Figure 1). Cells used in all the immunocytochemistry were fixed with 4% paraformaldehyde, and therefore, cell membranes of all 5 UM cell lines were permeabilized. This allowed the CXCR4 anti-body to stain internalized cytoplasmic CXCR4 receptor complexes. This occurs due to a response to ligand stimulation, where the chemokine receptors will undergo endocytosis mediated by clathrin-coated vesicles [19].

### Proliferation assay

**Figure 2:**
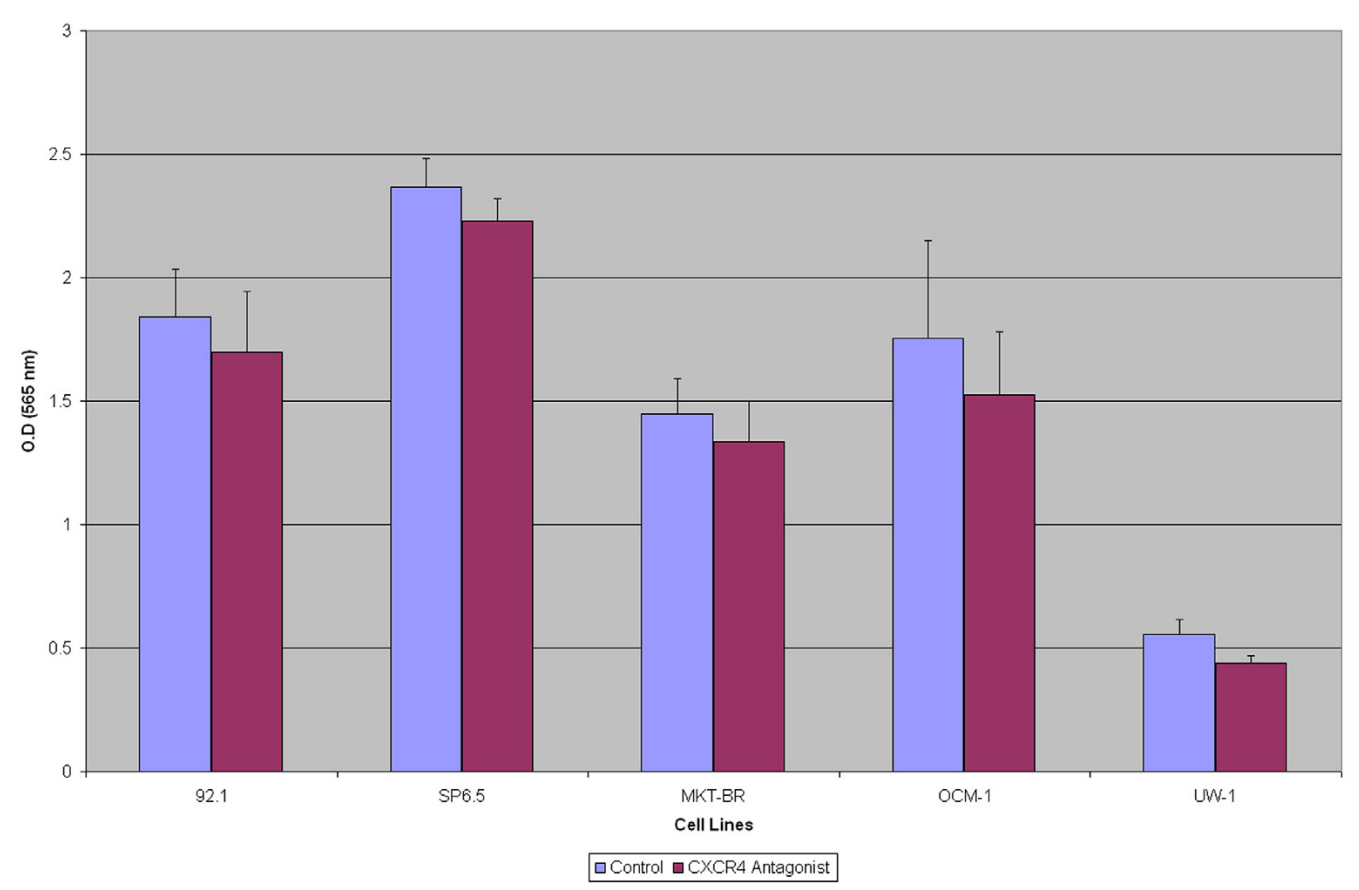
**The effect of TN14003 on UM cellular proliferation**. A bar graph depicting unaffected cellular proliferation of five UM cell lines when incubated with TN14003 (p > 0.05).

The proliferation rate of the 5 UM cell lines when pre-incubated with TN14003 did decrease slightly in all 5 cell lines, but was not statistically significant (p > 0.05) when compared back to the lines that were not pre-incubated with the peptide inhibitor (Figure 2).

### Migration assay

**Figure 3:**
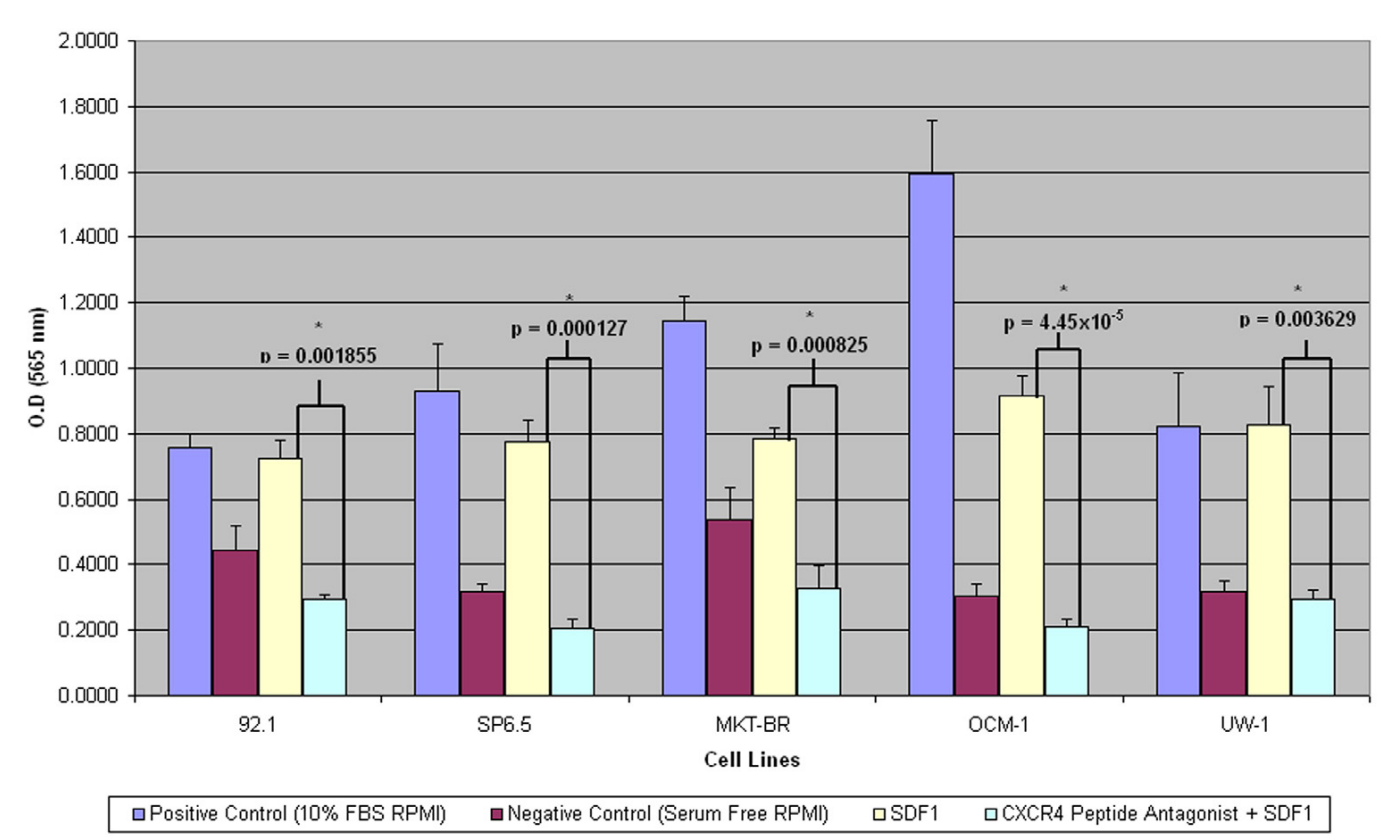
**Migratory inhibition of UM cell lines using TN14003**. A bar graph depicting the inhibition of cellular migration for five UM cell lines when incubated with TN14003 (p < 0.01).

All cell lines migrated towards the chemokine CXCL12 (200 ng/ml) at a level greater than the negative control (p < 0.05). All 5 cell lines pre-incubated with TN14003 (4 ng/ml) did not migrate towards the chemokine CXCL12 (p < 0.01 in all cases). A student's t-test was used in order to ascertain a significant change between groups that contained the inhibitor and the groups that did not contain the inhibitor. The 10% FBS (positive control) gave the highest levels of migration for each cell line. The serum-free medium (negative control) caused little to no migration in all cases (Figure 3).

### Flow cytometry

Cell surface expression of CXCR4 on the 5 uveal melanoma cell lines was verified using Fluorokine: Cytokine Flow Cytometry Reagents for Human SDF-1α Biotin Conjugate (R&D Systems). All 5 cell lines expressed different relative levels of the cell surface receptor CXCR4 (Table 1).

**Table 1:** Relative values of mean fluorescent intensity of SDF-1α binding to surface CXCR4 receptors on 5 UM cell lines as measured by a flow cytometer.

**Cell Line**	**Negative Control**	**SDF-1α**	**TN14003 Inhibitor + SDF-1α**
**92.1**	5.51	346	309
**SP6.5**	5.08	174	148
**MKT-BR**	5.85	263	228
**OCM-1**	6.57	227	196
**UW-1**	6.97	239	184

We also analyzed if TN14003 (4 ng/ml) would bind to cell surface CXCR4 receptors and shift the binding of the biotinylated SDF-1α cytokine, in turn causing a shift in the level of florecesence when being read by the flow cytometer. A negative shift in overall fluorescence was observed for all 5 cell lines when pre-incubated with TN14003 (4 ng/ml) for 5 minutes prior to treatment with SDF-1α biotinylated cytokine (Table 1).

**Figure 4:**
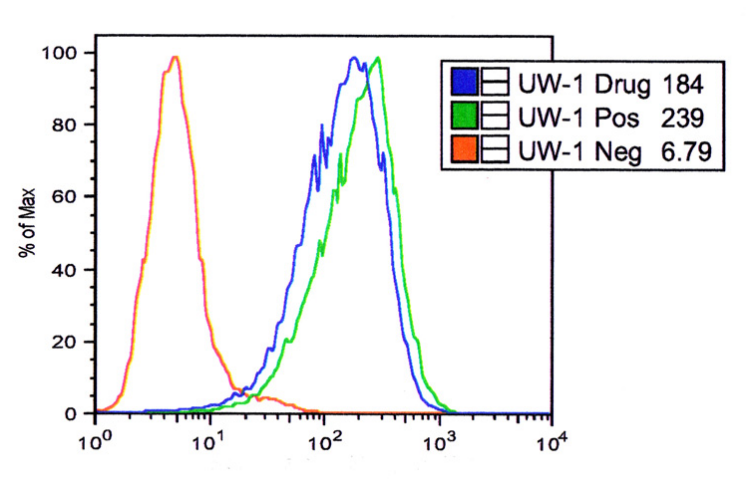
**Flow cytometric analysis of CXCR4 cell surface expression**. a) Green line displaying mean fluorescence of CXCR4 surface receptor density for human UM cell line UW-1 using a FITC-avidin complex for biotinylated SDF-1α. b) Blue line displaying a negative shift in mean fluorescence caused by pre-incubating UW-1 with TN14003 (4 ng/ml) before treatment with FITC-avidin complex.

We have also shown that TN14003, a CXCR4 peptide receptor antagonist, did indeed bind to the CXCR4 receptor shifting the binding of biotinylated ligand SDF-1α (CXCL12) in all 5 cell lines used. This proves active binding of the inhibitor which adversely shifted the mean fluorescent values of all 5 UM cell lines pre-incubated with the drug at a very low concentration (4 ng/ml) (Figure 4).

## Discussion

There has recently been an overwhelming amount of evidence suggesting that the CXCR4/CXCL12 chemokine signaling axis may play an integral role in the metastatic progression of many tumor types. It has been shown that CXCR4 may have a prognostic value when expressed in certain tumors such as esophageal cancer [20]. These authors have demonstrated that positive CXCR4 expression in the primary tumor post pre-operative chemoradiotherapy correlates very strongly with progression to distant metastasis, which provides clinicians with an independent prognostic factor for esophageal squamous cell carcinomas [20]. In non-small cell lung cancer it has been shown that the CXCR4 receptor, quantified by real-time PCR analysis, is present in higher concentration within patients that developed clinical metastasis than in those that did not develop metastasis [21]. Cutaneous melanoma immunohistocemical studies have also shown that CXCR4 is a predictor of poor prognosis [22]. Further studies have analyzed the expression of CXCR4 in metastasis of human cutaneous melanomas, and have conferred active CXCR4 expression and displayed the possibility of interfering with this axis in order to inhibit metastasis using a novel inhibitor AMD3100 [23]. We have revealed expression of a functional CXCR4 receptor in all 5 uveal melanoma cell lines that were studied. We have also previously shown that CXCL12 is not expressed in any these five cell lines via real-time PCR anaylsis [14]. This point is particularly interesting to note due to the fact that it has previously been shown in colorectal carcinoma that when endogenous sources of CXCL12 are epigenetically silenced, these cells more readily metastasize compared to those that engage in CXCR4/CXCL12 autocrine signaling [15]. This suggests that human uveal melanoma cell lines will be more likely to have a greater metastatic potential than other cell lines that actively produce CXCL12 thus creating an autocrine signaling loop.

The inhibitory potential of TN14003 a CXCR4 peptide antagonist has been previously studied in inhibiting breast cancer metastasis [12] and inhibiting the migratory and invasive ability of human pancreatic cancer cell lines [24]. Both studies concluded that TN14003 was a very effective inhibitor of the CXCR4/CXCL12 chemokine axis, and may be a possible future therapy for breast and pancreatic cancer. We showed that TN14003 at concentrations of 4 ng/ml is extremely effective at inhibiting the migratory ability of human uveal melanoma cell lines. TN14003 did not significantly reduce the proliferative ability of the cells due to the fact that CXCL12 is not produced by these cell lines. This provides further evidence that an autocrine loop is not active in these particular cell lines.

FITC-avidin kit for biotinylated SDF-1α displayed different relative surface expression of the CXCR4 receptor in the UM cell lines. Cells lines previously described as having a higher invasive and proliferative potential in vitro correlated with higher CXCR4 expression. For example, the 92.1 cell line has been previously characterized in vitro [25] and in vivo [26] as having a very high metastatic potential relative to other human UM cell lines such as SP6.5, MKT-BR, OCM-1, and UW-1.

Our study has shown that 92.1 does indeed have higher expression of surface CXCR4 relative to the other 4 UM cell lines used this experiment. This may implicate that CXCR4 may be necessary in higher concentration in order to achieve a greater metastatic potential. Knowing this, further in vivo studies should be preformed in order to substantiate the previously mentioned hypothesis.

## Conclusion

The characterization of the CXCR4/CXCL12 chemokine axis in five UM cell lines have shown significant reduction of cancer cell migration in vitro. We have also displayed that the TN14003 inhibitor binds CXCR4 with high affinity shifting the binding of its native ligand CXCL12. Cellular proliferation of the UM cell lines tested were not significantly inhibited by TN14003. This data indicates that further in vivo studies should be pursued in order to test TN14003 ability to inhibit UM metastasis.

## Authors' contributions

SD carried out the cellular migration assays, flow cytometry experiments, and wrote the manuscript. JCM and VBF preformed all proliferation assays and statistical analysis. EA and PL prepared all the cytospins and preformed all immunocytochemistry. BFF and MNB evaluated all immunocytological stainings, and revised the entire manuscript.
